# Fetal sex and maternal insulin resistance during mid-pregnancy: a retrospective cohort study

**DOI:** 10.1186/s12884-020-03242-x

**Published:** 2020-09-24

**Authors:** Hiroshi Yamashita, Ichiro Yasuhi, Megumi Koga, So Sugimi, Yasushi Umezaki, Misao Fukuoka, Sachie Suga, Masashi Fukuda, Nobuko Kusuda

**Affiliations:** Department of Obstetrics and Gynecology, NHO Nagasaki Medical Center, 1001-1 kubara 2-chome, 856-8562 Omura-City, Nagasaki, Japan

**Keywords:** Fetal sex, Maternal insulin resistance, Pregnancy

## Abstract

**Background:**

Recent studies have suggested that fetal sex influences maternal glucose and insulin metabolism during pregnancy. We examined whether fetal sex is associated with maternal insulin resistance and the β-cell function during mid-pregnancy.

**Methods:**

This retrospective study included singleton pregnant women who underwent a 75-g oral glucose tolerance test (OGTT) at 24–34 weeks of gestation due to positive diabetic screening. In addition to plasma glucose (PG), we measured plasma insulin during the OGTT to obtain surrogate indices associated with insulin resistance (IR), including homeostasis assessment model (HOMA) -IR and insulin sensitivity index (IsOGTT), and β-cell function, including insulinogenic index (II), HOMA-β, and area under the curve of insulin response. We compared these indices between women carrying male fetuses to those carrying female fetuses.

**Results:**

The study population included 617 women (mean age, 32.4 ± 4.9 years) with a mean pre-pregnancy body mass index (BMI) of 22.6±4.5. They underwent the 75g-OGTT at 29.0 ± 2.5 weeks. Two hundred fifty-eight (42%) women were diagnosed with gestational diabetes (GDM). There was no significant difference in maternal age, pre-pregnancy BMI, gestational age at OGTT, PG at OGTT, or the prevalence of GDM between women with a male fetus (*n*=338) (male group) and those with a female fetus (*n*=279) (female group). Regarding the indices of IR, IR was significantly higher and insulin sensitivity was lower in the female group than in the male group (HOMA-IR: 7.0 [5-9.6] vs. 6.2 [4.6-8.8], *p*< 0.05; IsOGTT: 5.86 [4.29-7.83] vs. 6.29 [4.59-8.84], *p*< 0.01) (median [quartile range]). These differences remained significant after adjustment for maternal age, pre-pregnancy BMI, gestational age and fasting PG at OGTT, and the diagnosis of GDM. In contrast, the β-cell function did not differ between the two groups.

**Conclusion:**

Maternal IR during mid-pregnancy was significantly higher in women carrying a female fetus than in those with a male fetus. The sex of the fetus may affect maternal insulin sensitivity during mid-pregnancy.

## Background

In perinatal medicine, sex differences are often observed, such as birthweight and the incidence of neonatal respiratory disorders. In addition, in maternal-fetal medicine, women carrying male fetuses have higher rates of fetal macrosomia, failure to progress during the first and second stages of labor, cord prolapse, nuchal cord, true umbilical cord knots, and cesarean section rate [1, 2]. These studies concluded that male sex is an independent risk factor for adverse pregnancy outcomes; however, the mechanisms by which fetal sex may contribute to these events are not clearly understood. Recently, Retnakaran et al. [3, 4] reported that women carrying a male fetus showed an increased risk of gestational diabetes (GDM), and that women with GDM who delivered a girl showed early progression of type 2 diabetes mellitus (T2DM) [3]. On the other hand, Xiao et al. [5] reported that compared with women carrying male fetuses, maternal insulin resistance was greater in those carrying female fetuses, although there was no sex difference in the development of GDM. Under the controversy, the aim of our study was to examine whether fetal sex is associated with maternal insulin resistance (IR) and β-cell function during mid-pregnancy in Japanese pregnant women.

## Methods

We conducted a retrospective study of singleton pregnant women who underwent a 75 g oral glucose tolerance test (OGTT) at 24–34 weeks of gestation because of a positive diabetic screen between January 2003 to October 2014 at the National Hospital Organization Nagasaki Medical Center. Universal screening of all pregnant women for GDM was performed with a 50-g glucose challenge test (GCT), and women with 1-hour value of ≥ 135 mg/dL underwent a diagnostic 75 g OGTT. Women with a GCT value of < 135 mg/dL but who had risk factors including maternal age ≥ 35 years, pre-pregnancy body mass index (BMI) ≥ 24 kg/m^2^, a history of previous GDM, and women with fetal macrosomia/polyhydramnios in the current pregnancy, also underwent a diagnostic OGTT. In addition to maternal basic characteristics and perinatal outcomes, we obtained the following data: plasma glucose (PG) and insulin values at fasting, 30-minute, 1-hour and 2-hour after an oral glucose load during 75 g-OGTT. We used two different diagnostic criteria to diagnose GDM during the study period: the Japan Society of Obstetrics and Gynecology (JSOG) criteria [6], which were used before June 2010, and the International Association of the Diabetes and Pregnancy Study Groups (IADPSG) criteria [7], which were used after July 2010. The JSOG criteria included cutoff PG values of 100, 180 and 150 mg/dL, at fasting, 1-hour and 2-hour after 75 g oral glucose load, respectively, and the IADPSG criteria included those values of 92, 180 and 153 mg/dL, respectively. By the former criteria, women were diagnosed as GDM when they met two or more abnormal PG values, and by the latter criteria, women with one abnormal value were diagnosed. To consider the effect of the different diagnostic criteria during the study period, we applied both the JSOG and the IADPSG criteria to the all subjects during the whole periods, and the results were categorized as GDM by the JSOG criteria (JSOG-GDM) which met the JSOG criteria without considering the IADPSG criteria, GDM by the IADPSG (IADPSG-GDM) which met the IAGDSG criteria but not the JSOG criteria, and non-GDM which revealed normal by both the criteria. We used the diagnostic category as a confound variable in multivariate analysis. We excluded women with overt diabetes during pregnancy according to the IADPSG criteria [7] because of the possibility of prepregnancy diabetes. We calculated surrogate indices of insulin resistance (IR), including homeostasis assessment model of IR (HOMA-IR) [8], insulin sensitivity index (IsOGTT) [9], and β-cell function indices including insulinogenic index (IGI) [10] and HOMA-β [8]. HOMA-IR was calculated as fasting plasma glucose (FPG) × fasting immunoreactive insulin (FIRI) / 405, and IsOGTT was calculated as 10,000 divided by the square root of (FPG × FIRI) × (mean glucose × mean insulin). To assess the function of insulin secretion, insulinogenic index (IGI) = (IRI at 30-min -FIRI) / (PG at 30-min - FPG), HOMA-β = (FIRI × 360)/(FPG-63), and area under the curve (AUC) of the total and the increment of IRI during 75 g-OGTT were calculated.

We compared these dynamic indices during pregnancy between women carrying a male fetus (the male group) and those carrying a female fetus (the female group). We also investigated whether the fetal sex is associated with the incidence of GDM. This study was approved by the Institutional Review Board of Nagasaki Medical Center with opt-out consent to obtain patient data from medical records.

We used Student’s *t*-test and the chi-squared test to compare variables between the groups. In order to assess to interactions between fetal sex and surrogate IR and β-cell function, we performed a multivariate regression analysis to control for confounding variables including maternal age, pre-pregnancy BMI, primiparity (yes/no), gestational age at OGTT, FPG value and the presence of GDM category. All statistical analyses were performed using the JMP9 software program (SAS Institute, Cary, NC, USA). *P* values of < 0.05 were considered to indicate statistical significance.

## Results

We included 617 Japanese singleton pregnant women; 338 (54.8%) women carrying a male fetus and 279 (45.2%) women carrying a female fetus. There was no significant difference in either the maternal characteristics or the metabolic data, including glucose and insulin values during the diagnostic OGTT, between the women in the male and female groups (Table 1). The prevalence of each GDM diagnostic category did not significantly differ between the fetal sex (Table 1).

**Table 1:** Comparison of maternal characteristics and the results of 75gOGTT at 24–34 weeks of gestation between women carrying a male fetus (the male group) and those carrying a female fetus (the female group)

	Male group (*n* = 338)	Female group (*n* = 279)	*P* value
Age (years)	32.5 ± 4.9	32.4 ± 5.0	0.75
Primiparity (%)	145 (43%)	122 (44%)	0.94
Pre-pregnancy BMI (kg/m^2^)	22.5 ± 4.2	22.7 ± 4.8	0.51
GA at OGTT (weeks)	29.0 ± 2.5	28.9 ± 2.5	0.34
FPG (mg/dl)	81.3 ± 8.6	81.6 ± 7.6	0.35
1hr-PG (mg/dl)	155.3 ± 31.2	158.5 ± 29.7	0.18
2hr-PG (mg/dl)	132.9 ± 28.4	133.5 ± 28.5	0.85
FIRI (µU/mL)	6.2 (4.6–8.8)	7.0 (5-9.6)	0.0090
1 h-IRI (µU/mL)	57.7 (38.1–86.7)	60.5 (42.1–86.9)	0.28
2 h-IRI (µU/mL)	53.1 (38.3–74.8)	52.1 (36.0-79.7)	0.86
HbA1c (%)	5.3 ± 0.5	5.4 ± 0.5	0.74
GDM (%)	138 (41%)	120 (43%)	0.63
JSOG-GDM	47 (14%)	43 (15%)	0.80
IADPSG-GDM	91 (27%)	77 (28%)	

Data are expressed as mean ± standard deviation, number (%), or median (quartile range)

*BMI *Body mass index, *GA *Gestational age, *OGTT *Oral glucose tolerance test, *FPG *Fasting plasma glucose, *PG *Plasma glucose, *FIRI *Fasting immunoreactive insulin, *IRI *Immunoreactive insulin, *GDM *Gestational diabetes, *JSOG *Japan Society of Obstetrics and Gynecology, *IADPSG *International Association of the Diabetes and Pregnancy Study Groups

The perinatal outcomes are shown in Table 2. Although the male infants were born significantly earlier in comparison to the females, there was no significant difference in the birthweight between the males and females. Regarding IR during pregnancy, the results of all the surrogate indices showed that women with female fetuses were significantly more insulin resistant than those with male fetuses (Table 3). Those differences remained significant after adjusting for maternal age, pre-pregnancy body mass index (BMI), gestational age and fasting PG at OGTT, and the GDM diagnosis (FIRI, p < 0.01; HOMA-IR, p = 0.01; IsOGTT, p < 0.01). In contrast, the β-cell function did not differ between the groups (Table 3).

**Table 2:** Comparison of the perinatal outcomes between the groups

	Male group (*n* = 338)	Female group (*n* = 279)	*P* value
GA at delivery (weeks)	38.8 ± 1.8	39.1 ± 1.4	< 0.01
Cesarean section (%)	112 (33%)	86 (31%)	0.55
Birthweight (g)	3,026 ± 481	2,982 ± 482	0.25
SD of birthweight	0.15 ± 1.1	0.13 ± 1.3	0.84
Heavy-for-date (%)	43 (13%)	39 (14%)	0.72

Data are expressed as mean ± standard deviation

*GA *Gestational age, *SD *Standard deviation

**Table 3:** Comparison of the indices of maternal insulin dynamics at 24–34 weeks of gestation between the groups

	Male group (*n* = 338)	Female group (*n* = 279)	Crude P value	Adjusted* P value
FIRI (µU/mL)	6.2 (4.6–8.8)	7.0 (5-9.6)	0.0090	0.0061
HOMA-IR	1.24 (0.89–1.86)	1.41 (1.01–1.98)	0.012	0.014
IsOGTT	6.29 (4.59–8.84)	5.86 (4.29–7.83)	0.025	0.0072
HOMA-ß	135 (97–181)	138 (105–198)	0.14	0.058
IGI	0.61 (0.40–0.90) (*n* = 174)	0.60 (0.41–0.92) (*n* = 142)	0.95	0.83
AUC of the total IRI (µU x hours/mL)	90 (62–127)	90 (67–128)	0.36	0.068
AUC of the increase of IRI (µU x hours/mL)	76 (52–111)	77 (55–109)	0.43	0.11

* Adjusted for maternal age, pre-pregnancy BMI, gestational age and fasting PG at OGTT, and GDM diagnosis category

Data are expressed as median (quartile range)

*FIRI *Fasting immunoreactive insulin, *HOMA-IR *Homeostasis assessment model–insulin resistance, *IsOGTT *Insulin sensitivity index, *IGI *Insulinogenic index, *AUC *Area under the curve, *IRI *Immunoreactive insulin

## Discussion

Although we did not find any association between the fetal sex and the prevalence of GDM in the mothers in this retrospective study, we demonstrated that—during mid- and late pregnancy—women carrying a female fetus had higher insulin resistance, after adjustment for the considerable confounders.

Di Renzo et al. [2] reviewed the association between fetal sex and pregnancy outcomes and described that maternal GDM and fetal macrosomia more frequently occurred as complications in women carrying a male fetus than those carrying a female fetus. In a systematic review and meta-analysis of observation studies to investigate whether the maternal risk of GDM was associated with fetal sex differences [11], the authors concluded that pregnant women carrying a boy have a 4% higher relative risk of GDM than those carrying a girl. In contrast, Xiao et al. [5] found no association between the risk of GDM and fetal sex, which is similar to the findings of the present study. Indeed, in the meta-analysis by Jaskolka et al. [11], only 6 of 21 studies showed a significant difference according to the fetal sex. These inconsistent results may be caused by the difference in the sample size. Indeed, In the six studies, with the exception of one study with a small sample size (*n* = 439), the populations of all of the studies were > 25,000. Racial diversity may also have affected the results in the studies with a small sample size. In our study, despite the small sample size, the study population was homogeneous, because we only included women of Japanese ethnicity. Thus, our study might have had sufficient power to detect statistical significance.

A few studies have reported an association between fetal sex and maternal insulin dynamics. Retnakaran et al. [3] reported that in pregnant women, although the surrogate indices of insulin sensitivity, including HOMA-IR and IsOGTT, did not differ according to the sex of the fetus, the β-cell function, as measured by the insulinogenic index/HOMA-IR, was lower in women carrying a male fetus. More recently, Geng et al. [12] reported that the presence of a male fetus was an independent risk factor for elevated FPG and lower HOMA-β in Chinese mothers with normal glucose tolerance at 24–28 weeks of gestation. However, they did not find any difference in the FIRI and HOMA-IR values of the groups. These two studies [3, 12] concluded that women carrying a male fetus are at risk for abnormal insulin dynamics during pregnancy. On the contrary, similarly to our study, Xiao et al. [5] found that women carrying a female fetus had higher insulin resistance than those carrying a male fetus, and the difference remained significant after adjusting for confounders. Retnakaran et al. [4] investigated the association between fetal sex in women with GDM in their first pregnancy and the risk of recurrence of GDM in their subsequent pregnancy. They found that fetal sex in the first pregnancy did not affect the risk of recurrence in the second pregnancy. Interestingly, however, a female in the first pregnancy was associated with the development of T2DM before the subsequent pregnancy [4].

The mechanism underlying the effects of sex difference in fetus on the maternal glucose and insulin metabolism remains unexplained. The increase in maternal insulin resistance during late mid-pregnancy is a physiological change that reflects normal fetal growth acceleration during the period [13]. Pregnancy-associated hormones and adipokines, including placental lactogen, estrogen, leptin and tumor necrosis factor-α—which are mainly produced by placenta—increase with advancing gestation, especially during mid-pregnancy. These placental products are considered to cause enhanced maternal insulin resistance during mid-pregnancy [14], although the mechanism is not completely understood. Regarding the association between placental products and fetal sex, controversial results have been reported in the literature. It has been reported that mothers carrying a female fetus have higher levels of placental lactogen and estrogen [15, 16], which promote insulin resistance [17–19]. Another study [20] reported that maternal leptin levels were higher in women carrying a female fetus. On the other hand, Retnakaran et al. [3] failed to demonstrate an association between fetal sex and maternal adipokines (including leptin and adiponectin levels) or lipid concentrations (including total cholesterol and triglyceride). From the aspect of genetics, some studies have suggested an association between fetal sex and the maternal glycemic status during pregnancy [21–23]. These studies suggested that interaction between fetal sex and maternal polymorphism in progesterone receptor [21], angiotensin converting enzyme [22], and peroxisome proliferator-activated receptor gamma2 [23] may affect the maternal glycemic status during pregnancy.

The present study was associated with some limitations. First, in addition to the small sample size in the study, we evaluated maternal insulin resistance using surrogate indices, including HOMA-IR, and IsOGTT, because of the retrospective study design. Although the euglycemic glucose clamp method is gold standard for the assessment of insulin resistance, HOMA-IR and IsOGTT are considered to be crucially associated with the clamp method results [24]. The application of the clamp method in the clinical setting was impractical in the present study, which included 600 pregnant women. For the same reason, previous investigations [3, 5, 12] also used these surrogate indices. In addition, because of the retrospective study design and the limited sample size, it could not be concluded whether or not fetal sex was associated with maternal β-cell function. Finally, as we excluded women with a negative GCT result from this study, our results may not have reflected those of the whole population of Japanese pregnant women.

## Conclusions

In Japanese women at high risk for GDM who were carrying a female fetus maternal insulin resistance during mid-pregnancy was significantly higher than in those carrying a male fetus. The elevated insulin resistance in women with a male fetus was independent of age, pre-pregnancy BMI, and gestational age at testing. Although the mechanism through which sex affected the maternal insulin dynamics is unexplained, the sex of the fetus may affect maternal insulin sensitivity during mid-pregnancy.

## Data Availability

The datasets used and/or analyzed during the current study are available from the corresponding author on reasonable request.

## Abbreviations

BMI: Body mass index
FIRI: Fasting immunoreactive insulin
FPG: Fasting plasma glucose
GA: Gestational age
GCT: Glucose challenge test
GDM: Gestational diabetes
HOMA-IR: Homeostasis assessment model–insulin resistance
IADPSG: International Association of the Diabetes and Pregnancy Study Groups
IGI: Insulinogenic index
IR: Insulin resistance
IRI: Immunoreactive insulin
IsOGTT: Insulin sensitivity index
OGTT: Oral glucose tolerance test
PG: Plasma glucose
SD: Standard deviation
T2DM: Type 2 diabetes mellitus
